# Induction of potassium channel regulator KCNE4 in a submandibular lymph node metastasis model

**DOI:** 10.1038/s41598-022-15926-9

**Published:** 2022-08-01

**Authors:** Ryosuke Mano, Tomoko Tanaka, Shiho Hashiguchi, Hiroyuki Takahashi, Naoaki Sakata, Seiji Kondo, Shohta Kodama

**Affiliations:** 1 Department of Oral and Maxillofacial Surgery, Faculty of Medicine, Fukuoka University, Fukuoka, Japan; 2 Department of Regenerative Medicine and Transplantation, Faculty of Medicine, Fukuoka University, Nanakuma 7-45-1, Jonan-ku, Fukuoka, Japan; 3 Department of Gastroenterological Surgery, Faculty of Medicine, Fukuoka University, Fukuoka, Japan

**Keywords:** Cancer, Metastasis

## Abstract

Cancer cells often metastasize to the lymph nodes (LNs) before disseminating throughout the body. Clinically, LN metastasis correlates with poor prognosis and influences treatment options. Many studies have shown that cancer cells communicate with immune and stromal cells to prepare a suitable niche for metastasis. In this study, mice were injected with B16–F10 murine melanoma cells to generate a tongue submandibular lymph node (SLN) metastasis model in which genes of interest could be investigated. Microarray analyses were performed on SLNs, identifying 162 upregulated genes, some of which are known metastasis genes. Among these upregulated genes, *Kcne4*, *Slc7a11*, *Fscn1*, and *Gadd45b* were not associated with metastasis, and increased expression of *Kcne4* and *Slc7a11* was confirmed by real-time PCR and immunohistochemistry. The roles of KCNE4 in chemokine production and cell adhesion were examined using primary lymphatic endothelial cells, and demonstrated that *Ccl17* and *Ccl19*, which are involved in melanoma metastasis, were upregulated by KCNE4, as well as *Mmp3* matrix metalloproteinase. Expression of KCNE4 was detected in human LNs with metastatic melanoma. In conclusion, we found that LN metastatic melanoma induces KCNE4 expression in the endothelium of LNs.

## Introduction

Cancer cells, including melanoma cells, often metastasize through the lymphatic system to the regional lymph nodes (LNs) before spreading throughout the body via the bloodstream^1–6^. The presence of LN metastasis in cancer patients is correlated with poor prognosis and is often a factor in determining treatment strategies^7–10^. Therefore, to understand the mechanisms of LN metastasis, many animal models and clinical studies have been conducted. It has been reported that cancer cells communicate with immune cells and stromal cells locally and at metastatic sites, remodeling an environment that supports metastasis even before the tumor reaches secondary organs^11,12^. To prepare pre-metastasis niches, cancer cells use several factors, including cytokines, chemokines, extracellular matrix, microRNA, exosomes, and small extracellular vesicles^13–16^. The mechanism by which melanoma metastasizes to LNs has been reported to cause changes in sentinel LNs, such as increased lymphangiogenesis^17^ and induction of an immunosuppressive environment^18^.

According to previous clinical and animal model studies, the dissemination of cancer cells from the primary tumor to distant sites often occurs earlier than the diagnosis of the primary tumor^19–24^. Understanding the changes in the LNs before metastatic dissemination is critical for deciphering the first steps in the spread of tumor metastasis and for developing a therapeutic approach to prevent LN metastasis^25^. In this study, we established a metastasis model in which melanoma cells metastasize from the mouse tongue to the submandibular lymph nodes (SLNs) and analyzed early changes in gene expression in the SLNs.

## Results

### Development of a tongue SLN metastasis model

To develop a tongue SLN metastasis model, we transplanted mice with B16–F10^26^, a LN metastatic murine melanoma cell line, into the right side of the tongue (Supplementary Fig. S1A). The tumor size increased in accordance with the number of transplanted cells and with time after transplantation (Supplementary Fig. S1B, C). The rates of metastasis to SLNs on days 3, 7, 10, and 14 of mice injected with 1 × 10^5^ B16–F10 were 10%, 10%, 50%, and 100%, respectively (Supplementary Fig. S1D). In addition, metastasis to the lung was detected in 2 of 10 mice on day 14. Giemsa staining of SLNs on days 14 and 20 revealed melanoma melanin pigment (Supplementary Fig. S1E). These data showed that our tongue SLN metastatic model exhibited definite SLN metastasis by day 14 following injection with 1 × 10^5^ B16–F10 cells.

### Changes in gene expression levels in the early stage of SLN metastasis

**Figure 1:**
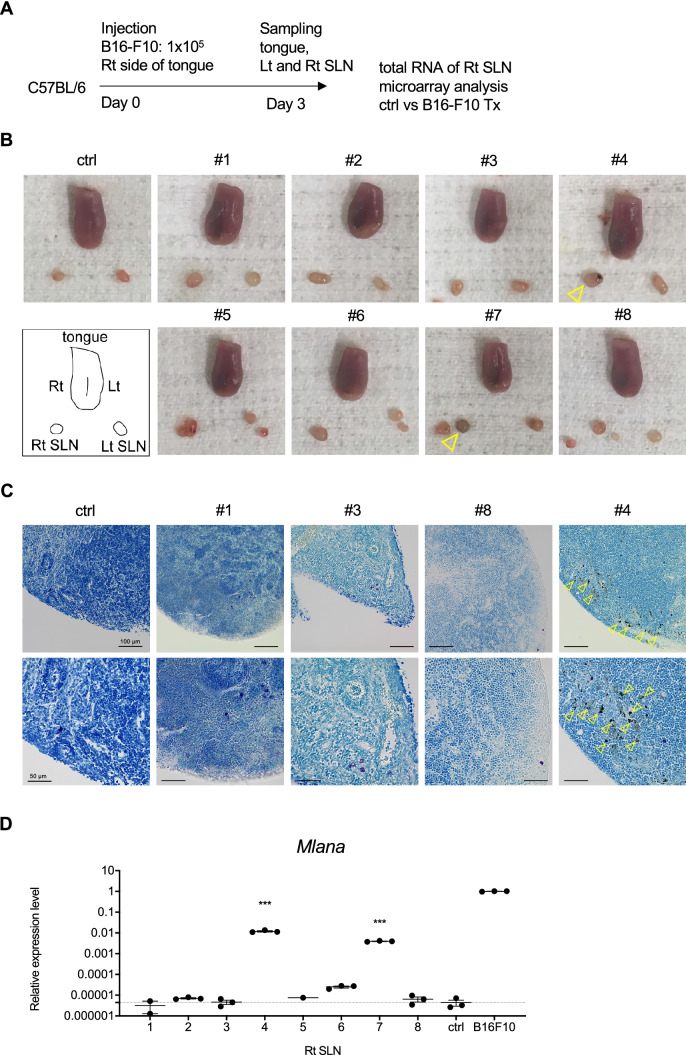
Selection of SLNs before melanoma metastasis for the analysis of early metastasis. (**A**) Schedule of sample preparation for microarray analysis. (**B**) Macroscopic images of the tongue and SLNs on day 3 after transplantation. Ctrl is a mouse injected with PBS and #1–8 are mice injected with B16–F10 into the tongue. LNs in which melanoma had metastasized turned black, as indicated by the arrowheads. (**C**) LNs #1, 3, and 8 with no metastasis and #4 with metastasis in macroscopic images were stained with Giemsa. Melanin pigment was detected as shown by the arrowhead in #4. Bars: 100 µm and 50 µm. (**D**) *Mlana* expression was examined by qRT-PCR in the right-hand LNs of mice #1–8 transplanted with B16–F10. Relative expression levels were adjusted for *Actb* expression. One-way ANOVA, ***p < 0.001 versus ctrl.

Next, to investigate gene expression changes of SLNs in the early stage of metastasis, we performed a microarray analysis. Eight mice were injected with B16–F10 and three with PBS into the right side of the tongue. Three days after the injection, gross observation revealed swelling of the right SLNs, and melanin pigmentation was observed in two (#4 and 7) of eight mice (Fig. 1A,B). The right SLNs were stained with Giemsa, and black melanoma cells were detected in the subcapsular sinus of #4, but not in the control or #1, 3, and 8 (Fig. 1C). To rule out SLNs in which B16–F10 had spread, the expression of a melanoma marker, *Mlana*, which encodes Melan A, was analyzed by quantitative RT-PCR (qRT-PCR), and the right SLNs of #1, 3, and 8 were selected for microarray analysis (Fig. 1D). SLNs from mice injected with PBS were used as controls.

**Figure 2:**
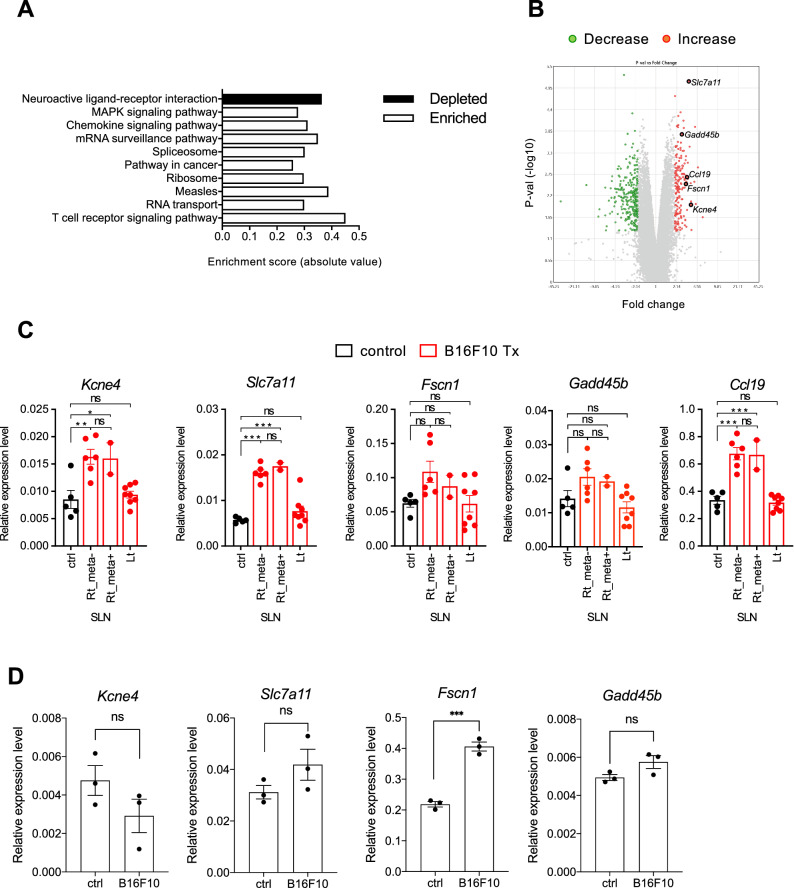
Genes with altered expression in SLNs during early metastasis. (**A**) Annotation analysis of genes whose expression had changed (B16–F10 vs control, fold change of < − 2 and > 2, respectively; *p* < 0.05). (**B**) Volcano plot of genes with altered expression. (**C**) Verification of *Kcne4, Slc7a11, Fscn1*, and *Gadd45b* expression by qRT-PCR. The expression levels of target genes in SLNs of control mice and SLNs of mice implanted with B16–F10 (right and left) were analyzed. One-way ANOVA, *p < 0.05, **p < 0.01, ***p < 0.001. ns; not significant. (**D**) LECs were co-cultured with B16–F10 in transwell plates for 24 h, and the expression levels of *Kcne4, Slc7a11*, *Fscn1 and Gadd45b* were analyzed by qRT-PCR. ***p < 0.001. ns; not significant.

In the results of the microarray analyses, there were 162 upregulated and 161 downregulated genes (B16–F10 vs control, fold change of < − 2 and > 2, respectively; *p* < 0.05). According to the annotation analyses, the upregulated genes were classified into the categories of T-cell receptor signaling pathway, RNA transport, and measles by annotation analysis, and the downregulated genes were classified as neuroactive ligand–receptor interactions (Fig. 2A). As expected, the upregulated genes included genes such as *Ccl19* that have been reported to be associated with metastasis (Fig. 2B). Among the upregulated genes, we focused on potassium voltage-gated channel, lsk-related subfamily, gene 4 (*Kcne4*), solute carrier family 7 (cationic amino acid transporter, γ+ system), member 11 (*Slc7a11*)^27,28^, fascin actin-bundling protein 1 (*Fscn1*)^29–31^, and growth arrest and DNA-damage-inducible 45 beta (*Gadd45b*)^32^, whose roles in LNs and metastasis are unknown. To validate the upregulation of these genes, we performed qRT-PCR, and compared their expression levels among control SLNs, right-hand SLNs without metastasis, right-hand SLNs with metastasis, and left-hand SLNs. The expression levels of *Kcne4, Slc7a11*, and *Ccl19* in the right-hand SLNs with or without metastasis were increased compared with the control SLNs and the left-hand SLNs (Fig. 2C). The expression levels of *Kcne4, Slc7a11,* and *Ccl19* in the right SLNs did not change depending on the presence or absence of metastasis (Fig. 2C).

To clarify whether the expression of *Kcne4, Slc7a11, Fscn1*, *Gadd45b* and *Ccl19* were induced by B16–F10-secreted factors, we analyzed changes in gene expression in LECs by qRT-PCR after co-culture in transwell plates. *Kcne4*, *Slc7a11* and *Gadd45b* were unchanged by co-culture with B16–F10, *Fscn1* was significantly induced, and *Ccl19* was undetectable in primary cultured LECs (Fig. 2D).

**Figure 3:**
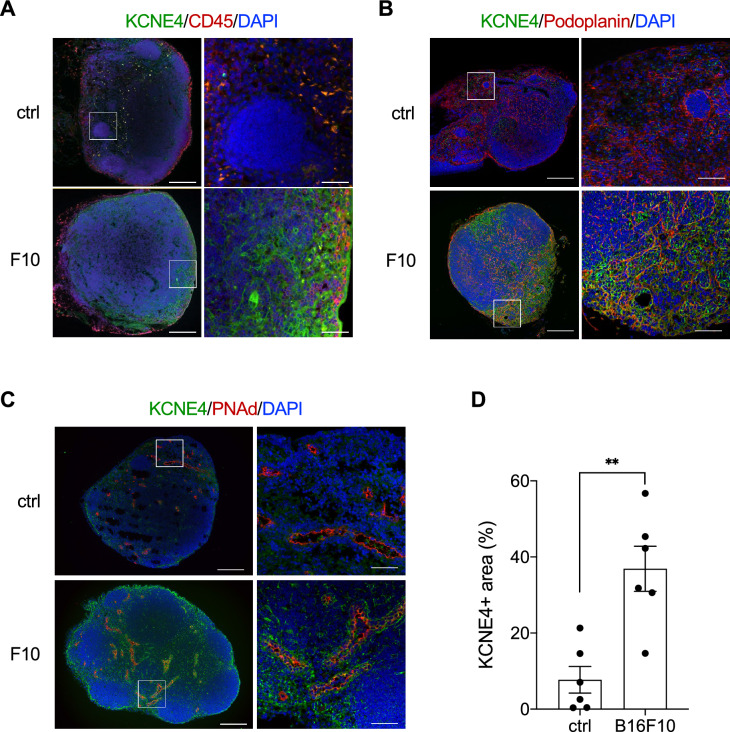
KCNE4 is upregulated by melanoma transplantation and is expressed on podoplanin-positive cells in SLNs. Expression of KCNE4 in SLN of mice transplanted with B16–F10 was examined by immunohistochemistry. Double staining was performed with anti-KCNE4 antibody and anti-CD45 antibody (**A**), anti-podoplanin antibody (**B**), and anti-PNAd antibody (**C**). Lymph nodes from mice injected with PBS were used as controls. Bars: 200 µm and 50 µm. (**D**) The percentages of KCNE4-positive areas in SLNs of ctrl or B16–F10 transplanted mice were measured by ImageJ software and divided by the area of the SLNs. Student’s *t* test, **p < 0.01.

Next, immunostaining was performed to determine which cells in the SLNs expressed the targets of interest. The expression levels of KCNE4, SLC7A11, and CCL19 were increased in the SLNs of B16–F10-transplanted mice compared with the controls (Fig. 3A–D, Supplementary Fig. S2A, B, G, and H). The KCNE4-positive ratios in the CD45-positive and podoplanin-positive areas of the SLNs of F10-transplanted mice were 15.2% and 62.1%, respectively (Fig. 3A,B). KCNE4 was also observed around PNAd-positive cells, a marker of high endothelial venules. (Fig. 3C). Most SLC7A11-positive cells were podoplanin-positive, whereas FSCN1-positive cells were negative for both podoplanin and CD45 (Supplementary Fig. S2C, D). GADD45B was observed in podoplanin-positive cells (Supplementary Fig. S2E, F). Consistent with previous reports, CCL19-expressing cells were podoplanin-positive (Supplementary Fig. S2G, H).

In addition to the mouse melanoma cell line, MOC2^33^ oral squamous cell carcinoma cells were modified to stably express turboGFP (MOC2-tGFP) and were transplanted into the tongue (Supplementary Fig. S3A). Seven days after transplantation, GFP fluorescence was detectable in the tongue but not in SLNs by stereomicroscopy (Supplementary Fig. S3B). The expression of tGFP in SLNs was detected by qRT-PCR (Supplementary Fig. S3C). *Kcne4* expression was detected in the right-hand SLNs of mice implanted with MOC2-tGFP (Supplementary Fig. S3D). *Kcne4* expression in B16–F10 and MOC2 cells was approximately one-thousandth of that in normal SLNs (Supplementary Fig. S3E).

### KCNE4 regulates the expression of chemokines and cell adhesion factors in primary cultured LECs

**Figure 4:**
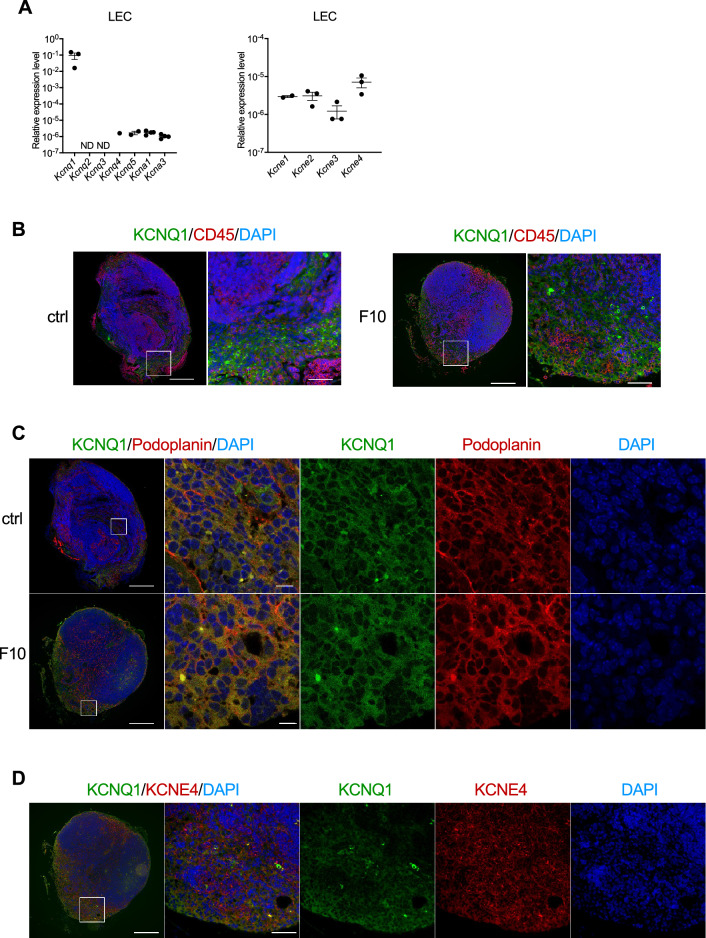
Expression of KCNQ1 and KCNE4 in SLNs. (**A**) Expression of *Kcnq* family members and *Kcna1* and *Kcna3* in primary cultured LECs were examined by qRT-PCR. The expression of KCNQ1 in SLNs was analyzed by immunostaining. Double staining of KCNQ1 and CD45 (**B**), Podoplanin (**C**), and KCNE4 (**D**) was performed. Bars: 200 µm and 50 µm (**B,D**), 200 µm and 10 µm (**C**).

We found that KCNE4 was induced in the SLNs of mice transplanted with B16–F10 and MOC2. KCNE4 is an inhibitory beta subunit of potassium voltage-gated channel subfamily Q member 1 (KCNQ1)^34–37^. KCNQ1 and KCNE4 are highly expressed in the heart^38,39^, but their roles in lymphatic endothelium are unclear. Because the mouse KCNQ family consists of KCNQ1, KCNQ2, KCNQ3, KCNQ4, and KCNQ5, we examined their expression levels by qRT-PCR and found that *Kcnq1* was dominantly expressed in LECs. (Fig. 4A). The mRNA expression of *Kcna1* and *Kcna3*, which also bind to KCNE4^40,41^, were detected whereas the expression levels of *Kcna1* and *Kcna3* were lower than *Kcnq1* (Fig. 4A). Among the KCNE family, *Kcne1*, *Kcne2*, *Kcne3*, and *Kcne4* were detected by qRT-PCR. (Fig. 4A). We examined whether KCNE4 was co-localized with KCNQ1 in SLNs. Immunostaining of SLNs of B16–F10 metastatic mice demonstrated that KCNQ1 was expressed in podoplanin-positive cells, but not in CD45-positive cells (Fig. 4B,C). In addition, KCNQ1 was detected in KCNE4-positive cells (Fig. 4D).

**Figure 5:**
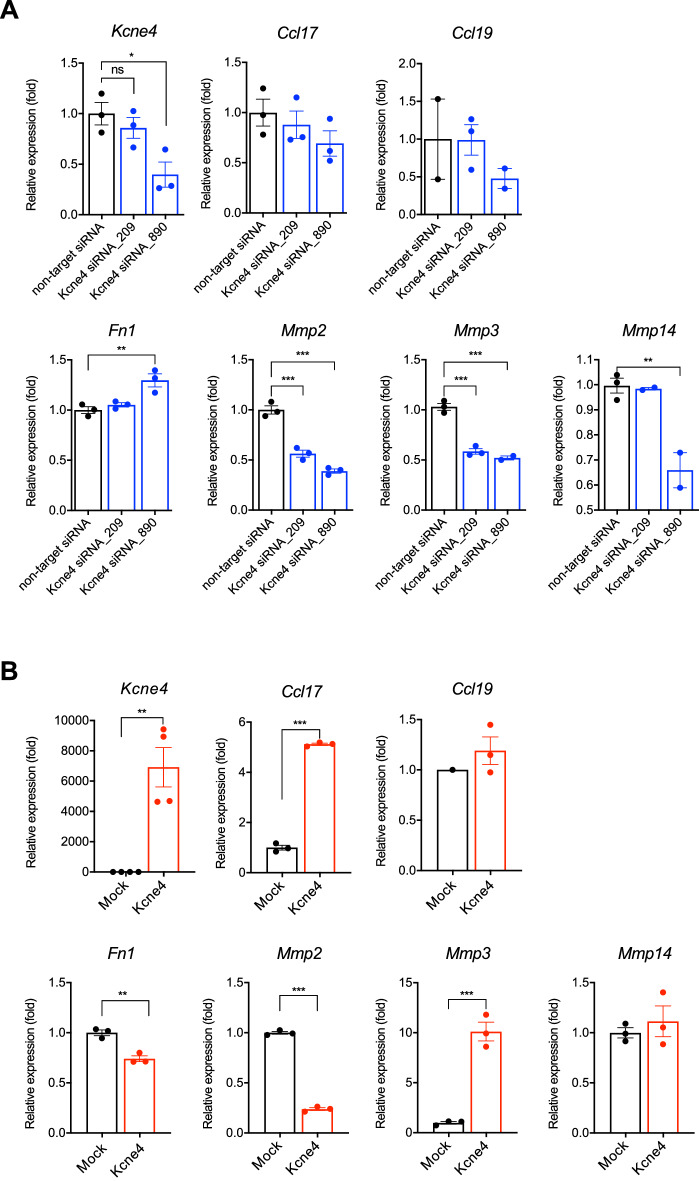
Effects of KCNE4 on the expression of chemokines and adhesion factors in primary cultured LECs. (**A**) *Kcne4* was suppressed by siRNA, and expression levels of *Ccl17, Ccl19, Fn1, Mmp2, Mmp3*, and *Mmp14* were analyzed by qRT-PCR. (**B**) LECs were transfected with KCNE4 expression plasmid, and expression levels of *Ccl17, Ccl19, Fn1, Mmp2, Mmp3*, and *Mmp14* were analyzed by qRT-PCR. One-way ANOVA, *p < 0.05, **p < 0.01, ***p < 0.001; Student’s *t* test, *p < 0.05, **p < 0.01, ***p < 0.001. ns; not significant.

Next, to investigate the roles of KCNE4 in lymphatic endothelium, *Kcne4* was suppressed by siRNA. In *Kcne4*-knocked down LECs, the expression of C-Cmotif chemokine ligand17 and 19 (*Ccl17* and *Ccl19*), which are involved in melanoma metastasis, was decreased. Conversely, overexpression of KCNE4 increased the expression of *Ccl17* and *Ccl19*, especially *Ccl17* (Fig. 5A,B). The adhesion factor fibronectin 1 (*Fn1*) was increased by knockdown of *Kcne4* and decreased by its overexpression (Fig. 5A,B). Regarding the metalloproteases, matrix metalloproteinases − 2, − 3, and − 14 (*Mmp2, Mmp3*, and *Mmp14*, respectively) were decreased by *Kcne4* knockdown, and overexpression of KCNE4 decreased *Mmp2* but markedly increased *Mmp3* (Fig. 5A,B).

### Expression of KCNE4 in clinical specimens of melanoma lymph node metastasis

**Figure 6:**
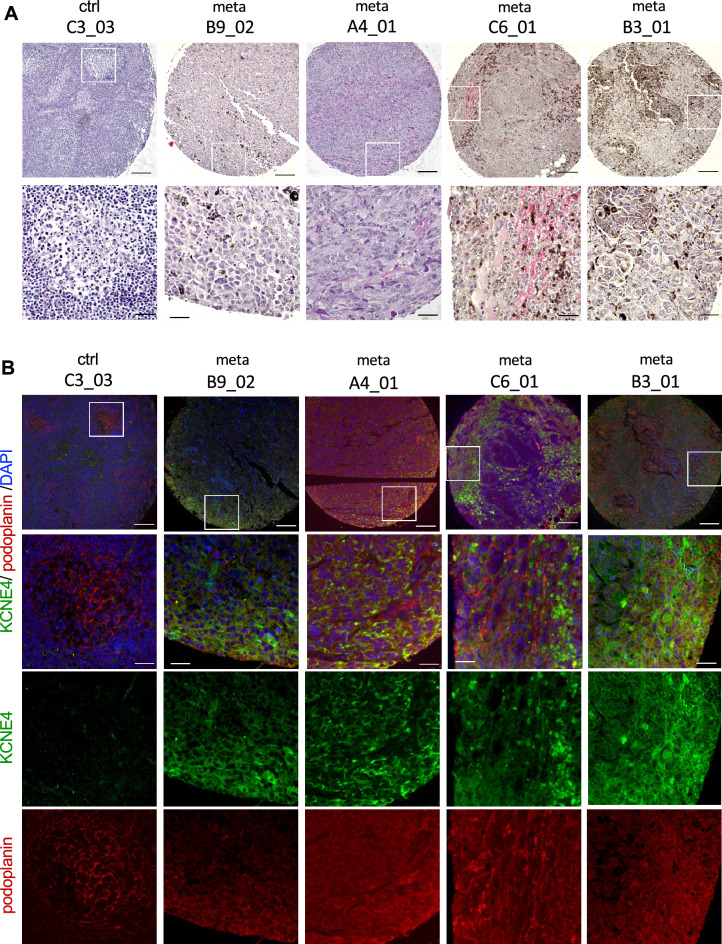
Expression of KCNE4 in human melanoma lymph node metastasis. (**A**) HE staining of human LNs with melanoma metastasis. (**B**) Human LNs were examined by immunostaining using anti-KCNE4 and anti-podoplanin antibodies. ctrl C3_03; normal LN. meta B9_02, A4_01, C6_01 and B3_01: metastatic malignant melanoma from the neck, groin, and neck and groin, respectively. Nuclei were stained with DAPI. Bars: 200 µm and 50 µm.

Immunostaining was performed to determine whether KCNE4, which was upregulated in the mouse melanoma metastasis model, was also expressed in human lymph node tissues to which melanoma had metastasized. As shown in Fig. 6, KCNE4 was detected in human LNs with metastasis, and the podoplanin-positive areas were KCNE4-positive.

## Discussion

We have developed a model in which cancer cells metastasize from the tongue to SLNs with high probability within a short period of time. We analyzed the gene expression changes in SLNs and found that *Kcne4* and *Slc7a11* were induced in lymphatic endothelium in the early stages of metastasis before cancer cells had metastasized.

The mouse B16 melanoma cell line is the most widely used tumor model and has been used to elucidate metastatic mechanisms as well as in the development of anticancer drugs. Genetically engineered models of melanoma development and metastasis have also been reported, including tyrosinase-specific *Braf* p.V600E/*Pten* knockout mice, which is a model of developing malignant melanoma in which antioxidant administration promotes LN metastasis^42,43^. Our model involves simple B16–F10 implantation in the tongue, which results in early and high rates of metastasis to SLNs. Because B16 is derived from cutaneous melanoma, this is not an orthotopic transplantation, but it is useful for studying tumor immunity related to LN metastasis because it is implanted into C57BL/6 mice in a syngeneic manner. We transplanted a mouse oral squamous cell carcinoma cell line, MOC2, into the tongue and confirmed metastasis to the SLNs as well as B16–F10.

To analyze the changes that occur in LNs before cancer cell metastasis, we analyzed the gene expression changes of SLNs in the early stages of metastasis in our model and found that *Kcne4* and *Slc7a11* were increased. In in vitro transwell cultures, co-culture with B16–F10 did not affect the expression of *Kcne4* and *Slc7a11*, suggesting that this was not a direct effect of the secreted factors of B16–F10. KCNE4 is a regulatory subunit of KCNQ1 and suppresses the KCNQ1 current in *Xenopus* oocytes and mouse cardiomyocytes^34,36^ Human *KCNE4* gene mutations are associated with a variety of pathologies, especially cardiac arrhythmias^39,44^. In studies of *Kcne4*-deficient mice, KCNE4 expression in ventricles was higher in males than in females, and was regulated by androgens^45^. In addition, KNCE4 is expressed in vascular smooth muscle^46^, but its function in lymphatic endothelium has not been reported. We found that KCNE4 was induced in podoplanin-positive cells in SLNs of B16–F10 transplanted mice. In primary cultured LECs, inhibition of KCNE4 decreased *Ccl17* and *Mmp3* and increased *Fn1*. Conversely, overexpression of KCNE4 increased *Ccl17* and *Mmp*3 and decreased *Fn1*, suggesting that KCNE4 promotes metastasis by increasing metastasis-associated cytokine production and lymphatic endothelial permeability. CCL17 is a ligand for CCR4, which activates CCR4-expressing Th2 cells and regulatory T-cells (Tregs) and suppresses effector cells; KCNE4 may contribute to tumor cell survival through activation of Tregs via increasing CCL17 production. MMP-3 (also known as stromelysin-1) has been reported to enhance the migratory and invasive abilities of tumor cells. The role of MMPs in cancers has been well elucidated, and they can remarkably promote the malignancy of tumor cells by degrading the extracellular matrix, facilitating angiogenesis, and promoting tumor invasion and metastasis^47^.

In leukocytes, KCNE4 has an important role in regulating KCNA3 (Kv1.3) to act as an inhibitor of cell proliferation, activation, apo-regulation, autoimmune diseases, and T cell proliferation and activation^40,41^. We showed that *Kcna3* expression was low in LECs and KCNE4 was induced in podoplanin-positive lymphatic endothelium in the early stages of metastasis. Although it is unclear how melanoma induces KCNE4 in lymphatic vessel endothelium, KCNE4 co-localized with KCNQ1 in LNs, suggesting that KCNE4 regulates KCNQ1 in lymphatic endothelium. Whether KCNQ1 expression has a functional role in the metastatic process remains unknown and will require further analysis.

SLC7A11, also known as xCT, is an amino acid exchanger that exports intracellular glutamate and imports cystine into the cell^27^. Cystine imported by xCT becomes a source of glutathione, which acts to remove reactive oxygen species^48^, suggesting that melanoma-inducing xCT in LECs could contribute to glutathione production in LECs.

We demonstrated that KCNE4 expression is also present in podoplanin-positive cells in human melanoma metastatic LNs. In the last decade, immune checkpoint inhibitors have been shown to be effective against malignant melanoma^49^, but they are less effective against mucosal melanoma than cutaneous melanoma^50^. The nasal cavity is the most common site of occurrence of malignant melanoma of the head and neck, followed by the oral cavity. However, the incidence of LN metastasis is significantly higher in the oral cavity (25%) than in the nasal cavity (5.7%)^51^. In the oral and maxillofacial region, cervical LN metastasis is present in 25% of patients at the time of initial diagnosis and in 42% of patients throughout the course of the disease, and is associated with poor prognosis^52^.

In conclusion, we established a model of tongue-submandibular LN metastasis and found that KCNE4 is increased in the LNs prior to metastasis. Further physiological studies are needed to analyze the role of KCNQ1–KCNE4 in lymphatic endothelium. To clarify the role of KCNE4 in metastasis, it will be necessary to transplant cancer cells into mice with LEC-specific deletion of *Kcne4* in the future.

## Methods

### Cell culture

C57BL/6 mouse skin melanoma cell line B16–F10 (CRL6475; ATCC, Manassas, VA)^26,53^ were cultured at 37 °C in a 5% CO_2_ incubator in DMEM (D5796; Sigma–Aldrich, St Louis, MO) supplemented with 10% fetal bovine serum (FBS) (Cytiva, Tokyo, Japan). The cells were used up to six passages in the experiments. C57BL/6 mouse-derived primary cultured LECs (Cell Biologics, Chicago, IL) were cultured in 0.1% gelatin-coated culture dishes at 37 °C with 5% CO_2_ in EGM2-endothelial cell growth medium-2 (Lonza, Basel, Switzerland), and used in in vitro experiments up to six passages^54^. For transwell culture, 5 × 10^4^ LECs were seeded into 24-well plates and incubated overnight. Transwell plates with 0.4-µm pore size (Corning, Corning, NY) were seeded with 1 × 10^5^ B16–F10 and co-cultured for 24 h. MOC2 mouse oral squamous cell carcinoma cell line was obtained from Kerafast (Boston, MA)^33^. Culture medium for MOC2 was a mixture of IMDM (Nacalai Tesque, Kyoto, Japan) and Ham's F12 (Nacalai Tesque) at a 2:1 ratio, supplemented with 5% FBS (Cytiva), 5 mg/L insulin (Sigma–Aldrich), 40 µg/L hydrocortisone (Sigma–Aldrich), and 5 µg/L EGF (R&D Systems, Minneapolis, MN). For stable expression of turboGFP, MOC2 were infected with GIPZ non-silencing lentiviral shRNA control (Horizon Discovery, Cambridge, UK) and cultured in medium containing 2 µg/mL puromycin (Sigma–Aldrich).

### Transplantation of melanoma cells into the tongue

All animal experiments were performed in compliance with the relevant laws and institutional guidelines and were approved by the Animal Care and Use Committee of Fukuoka University (approval number: 1810067), and in accordance with the ARRIVE guidelines. Eight-week-old male C57BL/6 mice were purchased from Kyudo (Tosu, Japan). Mice were kept under specific pathogen-free conditions and used at 9 weeks of age. B16–F10 or MOC2-tGFP cells were removed from the culture dish by trypsin treatment, and 1 × 10^4^, 1 × 10^5^, or 5 × 10^5^ cells were suspended in 50 µL PBS and injected into the right side of the tongue under anesthesia using 2% isoflurane. After transplantation, body weight was measured, and the tongue was observed macroscopically every 3 days. Mice that had lost more than 10% of their body weight in 1 week were killed with an overdose of isoflurane.

### Quantitative RT-PCR

Total RNA was prepared using a Purelink RNA Purification Kit (Thermo Fisher Scientific, Waltham, MA). Quantitative real-time PCR (qPCR) was performed using One-step TB Green Premix plus ExTaq II (Takarabio, Otsu, Japan) and a LightCycler96 (Roche Diagnostics, Basel, Switzerland). Primers used for qPCR are listed in Table S1. *Actb* was used as internal control, and expression levels were normalized to *Actb*.

### Microarray analysis

A total of 1 × 10^5^ of B16–F10 cells were transplanted into eight mice, and PBS was injected into three control mice. At 3 days after transplantation, LNs were removed and divided into two sections: one for qPCR, and the other for tissue staining. LNs with low expression levels of *Mlana* were used for microarray analysis. Sample labeling and array hybridization were performed on a Clariom D Assay, Mouse GE Microarray 8 × 60K v2 (Thermo Fisher Scientific). After quantile normalization, lncRNA was removed. Present probes were extracted and a Z score with a ratio was calculated. Enrichment analysis was performed by GeneTrail2 (https://genetrail2.bioinf.uni-sb.de/) and a volcano plot was created by Transcriptome Analysis Console software (TAC) (Thermo Fisher Scientific).

### Histological analyses

Mice were killed at each time point after cell transplantation, and the tongue and SLNs were removed, fixed in formalin, embedded in paraffin, and sliced to 4-µm thickness. Tissue sections were deparaffinized and stained with hematoxylin–eosin. Human lymph node tissue sections were obtained from US Biomax (Derwood, MD). Metastatic malignant melanoma tissue array (BCC38218) was used to stain melanoma metastatic LNs. Lymph node tissue array (LY481) was used for staining of control LNs. Immunohistochemistry was performed as described previously^54^. Primary antibodies used for immunohistochemistry are listed in Table S2. Secondary antibodies were Alexa Fluor 488-conjugated AffiniPure donkey anti-rabbit IgG, Alexa Fluor 594-conjugated AffiniPure goat anti-Syrian hamster IgG, Alexa Fluor 594-conjugated AffiniPure donkey anti-goat IgG, and Alexa Fluor 594-conjugated AffiniPure donkey anti-mouse IgG (all from Jackson ImmunoRessearch, West Grove, PA). Images were acquired using a fluorescence microscope (BZ-710; Keyence, Osaka, Japan) and a confocal microscope (LSM710; Carl Zeiss, Oberkochen, Germany). Image analysis was performed using Image J (https://imagej.nih.gov/ij/).

### Suppression and overexpression of KCNE4 in LECs

A total of 1.5 × 10^5^ LECs were transfected with 25 pmol non-targeting siRNA (MISSION siRNA universal control#1, MERCK, Darmstadt, Germany) or siRNA against *Kcne4* (Table S3) using Lipofectamine RNAiMAX (Thermo Fisher Science). Mouse *Kcne4* cDNA was cloned by PCR and inserted into pcDNA3.1 plasmid. pcDNA3.1-KCNE4 plasmid was transfected into LECs using Fugene HD (Promega, Madison, WI). Transfection was performed in accordance with the manufacturer’s instructions.

### Statistical analysis

Statistical analysis was performed using GraphPad Prism software ver.8 and 9. All data are expressed as the mean ± standard error. Comparative analysis was performed by Student’s *t* test or one-way analysis of variance (ANOVA). For multiple comparisons, we performed Bonferroni or Sidak analysis. The statistical significance was set at *p* < 0.05.

## Supplementary Information

Supplementary Information.

## Data Availability

Microarray data: https://www.ncbi.nlm.nih.gov/geo/query/acc.cgi?acc=GSE197190.
