# Supplementary material for: Induction of potassium channel regulator KCNE4 in a submandibular lymph node metastasis model
Source: Sci Rep. 2022 Aug 1;12:13208. doi: 10.1038/s41598-022-15926-9 (PMC9343410; doi:10.1038/s41598-022-15926-9)
Supplement: Supplementary file 1 — Supplementary Information. [file 41598_2022_15926_MOESM1_ESM.pdf]

Supplementary Information

Induction of potassium channel regulator KCNE4 in a submandibular lymph node metastasis model

Ryosuke Mano, Tomoko Tanaka, Shiho Hashiguchi, Hiroyuki Takahashi, Naoaki Sakata, Seiji Kondo, Shohta Kodama

Table S1. List of primers used for quantitative RT-PCR.

Target	GenBank Acc.	Sense 5'->3'	Antisense 5'->3'
Actb	NM_007393.5	CATCCGTAAAGACCTCTATGCCA	ATGGAGCCACCGATCCACA
Mlana	NM_029993.1	AGACGCTCCTATGTCACTGCTGAA	AGTACCAGCAGCCGATAAGCAGA
Kcne4	NM_021342.2	TTTGCTCGATGGAAGGGGAC	TCTCCTCCAGCTCGTCATCA
Slc7a11	NM_011990.2	CATCATCGGCACCGTCATCG	AGGACAGGGCTCCAAAAAGTG
Fscn1	NM_007984.2	CGCCAGGGAATGGACCTGT	GATTGCACACCTCCGGTCG
Gadd45b	NM_008655.1	TGCTCTTGGGGATCTTCCGT	CTGCATCTTCTGAACCGCGT
Ccl19	NM_011888.3	GTGTTCACCACACTAAGGGGC	TGTTGCCTTTGTTCTTGGCAGA
turboGFP	L29345.1	GCTTCTACCACTTCGGCACC	TCGTACTTCTCGATGCGGGT
Kcnq1	NM_008434.2	TTTGCCCGGAAGCCCATTTC	GCGGATACCCCTGATAGCTG
Kcnq2	NM_010611.3	GGCAGTACTACGAGCGGACA	GGCTCCTTCCTGAAGGTGAGT
Kcnq3	NM_152923.3	TGGGGCCTGATCACACTGGS	CCTGCCGGAAGGGCAAAA
Kcnq4	NM_001081142.3	GCGATAAGGGTCCTTCAGACAC	TCGATGGACTGCACCTGCTT
Kcnq5	NM_001160139.1	TCAGACACGCGTTGATCAAATTC	TGCTGGGGTCATCTGTTGTC
Kcna1	NM_010595.3	CCTTCAGATCCTGGGCCAGA	CCGCAAAGTACACTGCGCTA
Kcna3	NM_008418.2	CCATCGCAGGTGTCTTGACC	TCGGCTGAAGAGGAGAGGTG
Kcne1	NM_008424.3	GACCAGGCACCCAGAGTTTTG	GCCAGAAAGGGCAGAACAGT
Kcne2	NM_001358372.1	TTGCTCACATACCACACAGCAAGG	TTCCTCCTCCAGCTGTCCATA
Kcne3	NM_001360466.1	GGGGAGATGCCTGCTAGAGT	CCTGTTGTAGAGAGGCCCTG
Ccl17	NM_011332.3	TCCAGGGATGCCATCGTGTT	GCGTCTCCAAATGCCTCAGC
Fn1	NM_001276408.1	ATTCCCGAGGCATGTGCAG	ATTCCCGAGGCATGTGCAG
Mmp2	NM_008610.3	AGAGCGTGAAGTTTGGAAGCA	CCTGGTGTGCAGCGATGAAG
Mmp3	NM_010809.2	TCCTCCACAGACTTGTCCCG	CTTGGTGGGTACCACGAGGA
Mmp14	NM_008608.4	CTCACCCCAGCATTGCTTCA	GCTGAACACACACCGAGCTG

Table S2. List of siRNA sequences.

siRNA	Sequence 5’->3’	Complementary strand 5’->3’
non-target siRNA	UGGUUUACAUGUUUUCCUAUU	UAGGAAAACAUGUAAACCAUU
Kcne4 siRNA #1	AAUAUAGAAGUAUUCAUUGCC	CAAUGAAUACUUCUAUAUUUU
Kcne4 siRNA #2	AAAAAACACCACAUUAUCCAG	GGAUAAUGUGGUGUUUUUUCA

Table S3. List of antibodies used for immunohistochemistry.

Target	Antibody	Supplier / #
Podoplanin	Purified anti-mouse Podoplanin Antibody	Biolegend / 127401
CD45	Mouse CD45 Antibody	R&D Systems / AF114
KCNE4	Anti-KCNE4 antibody	abcam / ab254642
SLC7A11	Anti-xCT antibody	abcam / ab37185
FSCN1	Anti-Fascin antibody [EP5902]	abcam / ab126772
GADD45B	Anti-GADD45B antibody-C-terminal	abcam / ab230646
CCL19	MIP3 beta antibody	Biobyt / orb256345
KCNQ1	KCNQ1 antibody (G-8)	Santa Cruz Biotechnology / sc-365186
PNAd	Purified anti-mouse/human PNAd antibody	Biolegend / 120801
Podoplanin	Human Podoplanin Antibody	R&D Systems / AF3670

Supplementary Fig. S1

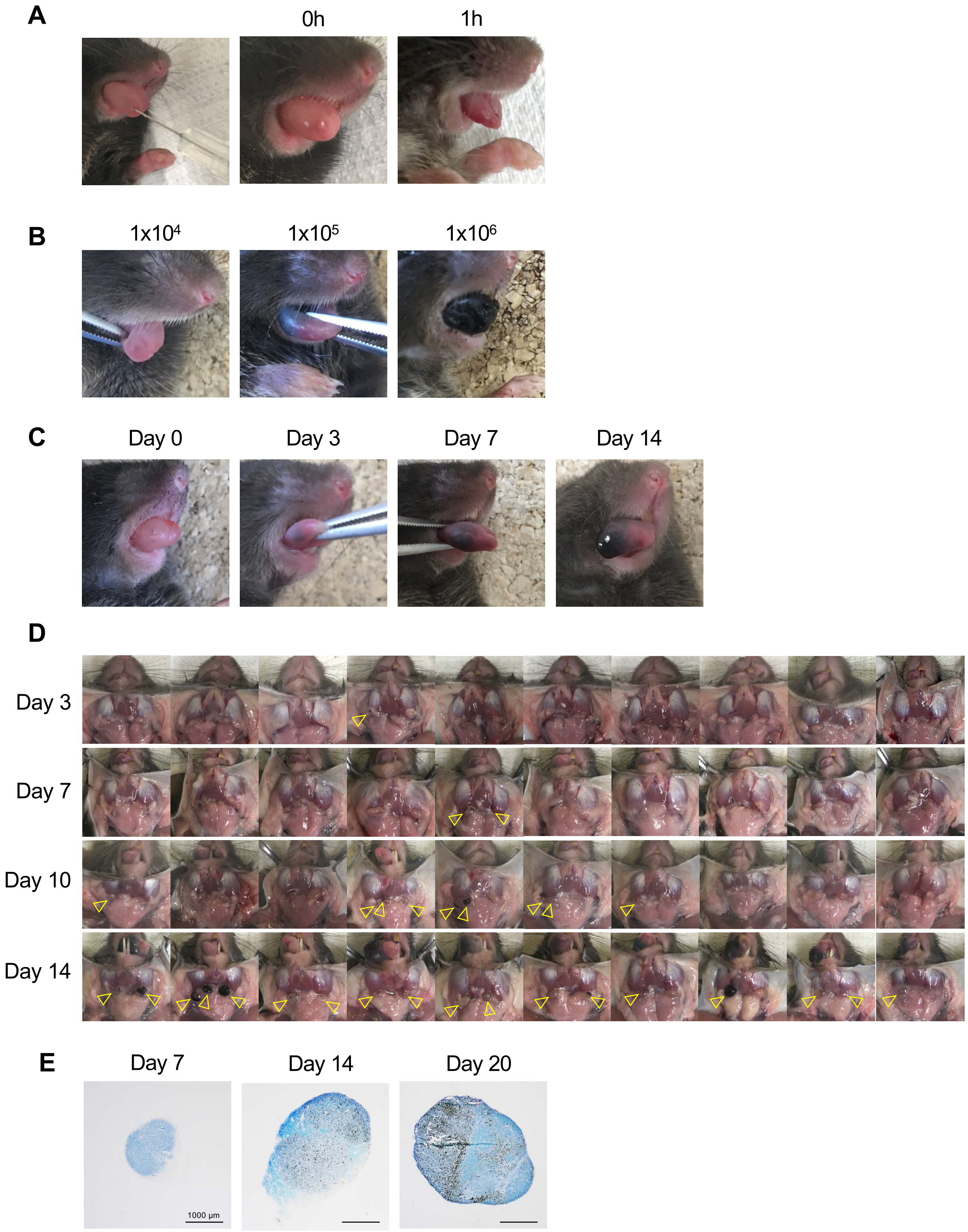

Supplementary Fig. S1. Transplantation of mouse melanoma cell line B16-F10 into the tongue and metastasis to submandibular lymph nodes. (**A**) Cells were injected into the right side of tongue. (**B**) Macroscopic image of the tongue on day 7 after transplantation. Mice were injected with 1×10^4, 1×10^5 and 1×10^6 of B16-F10. (**C**) Tumor growth in tongue transplanted with 1×10^5 cells was observed at 3, 7, 10 and 14 days. (**D**) Macro images of SLNs transplanted with B16-F10. The arrowheads indicate the SLNs where melanin pigment was observed. (**E**) Giemsa-stained image of SLNs. Melanin pigment deposition was detected and metastasized B16-F10 cells are observed in LNs at 14 and 20 days after transplantation. Bars; 1 mm.

Supplementary Fig. S2

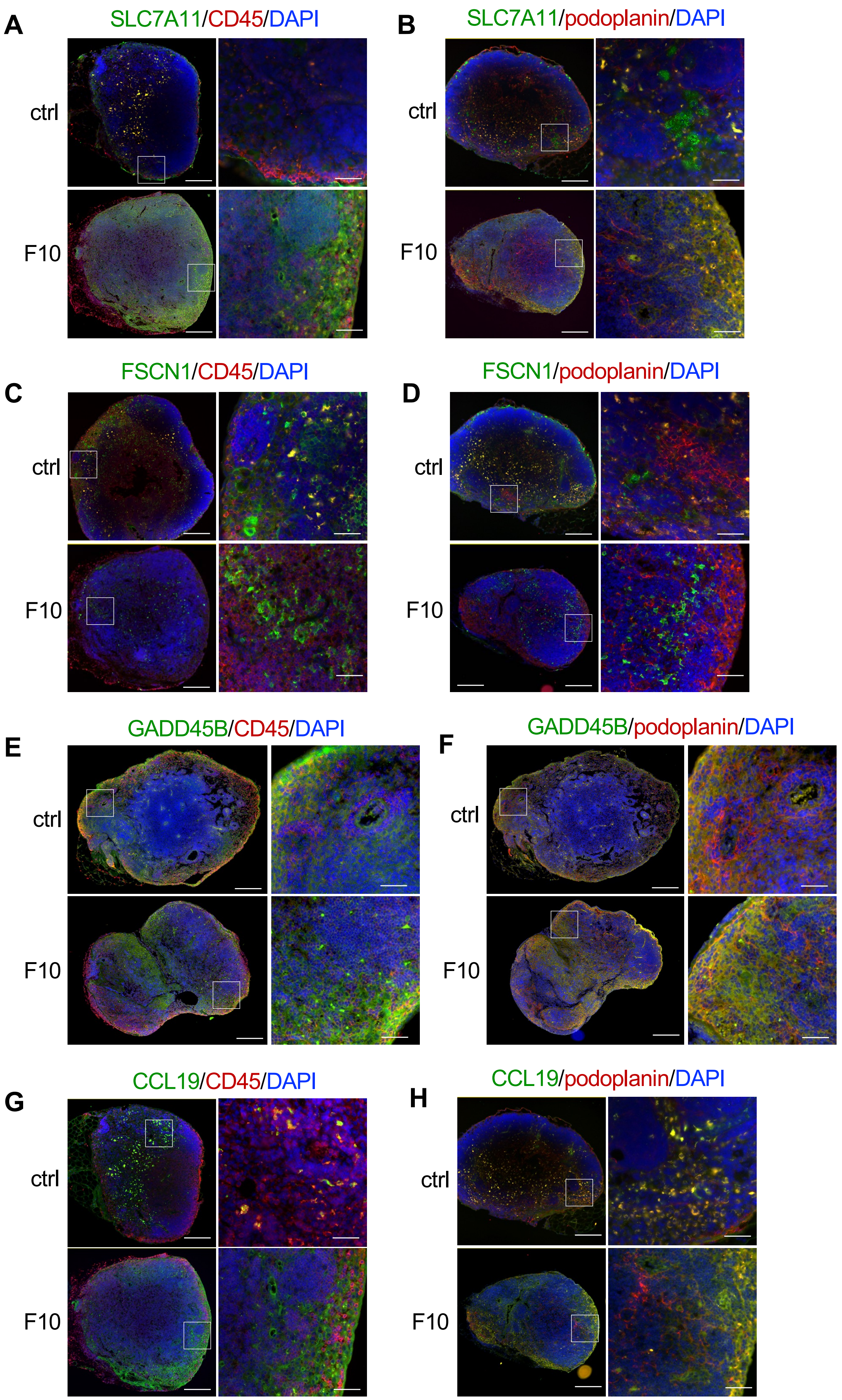

Supplementary Fig. S2. Expression of SLC7A11, FSCN1, GADD45B and CCL19 in SLN of B16-F10-transplanted mice. Among the genes up-regulated by B16-F10 transplantation, the expression of SLC7A11 (**A, B**), FSCN1 (**C, D**), GADD45B (**E, F**), and CCL19 (**G, H**) was examined by immunostaining. Double staining was performed for the pan-leukocyte marker CD45 and targets (**A, C, E, G**); double staining for podoplanin and targets (**B, D, F, H**). Ctrl is the SLN of mice injected with PBS on the tongue; F10 is the SLN of mice implanted with B16-F10 on the tongue. Bars; 200 μm and 50 μm.

Supplementary Fig. S3

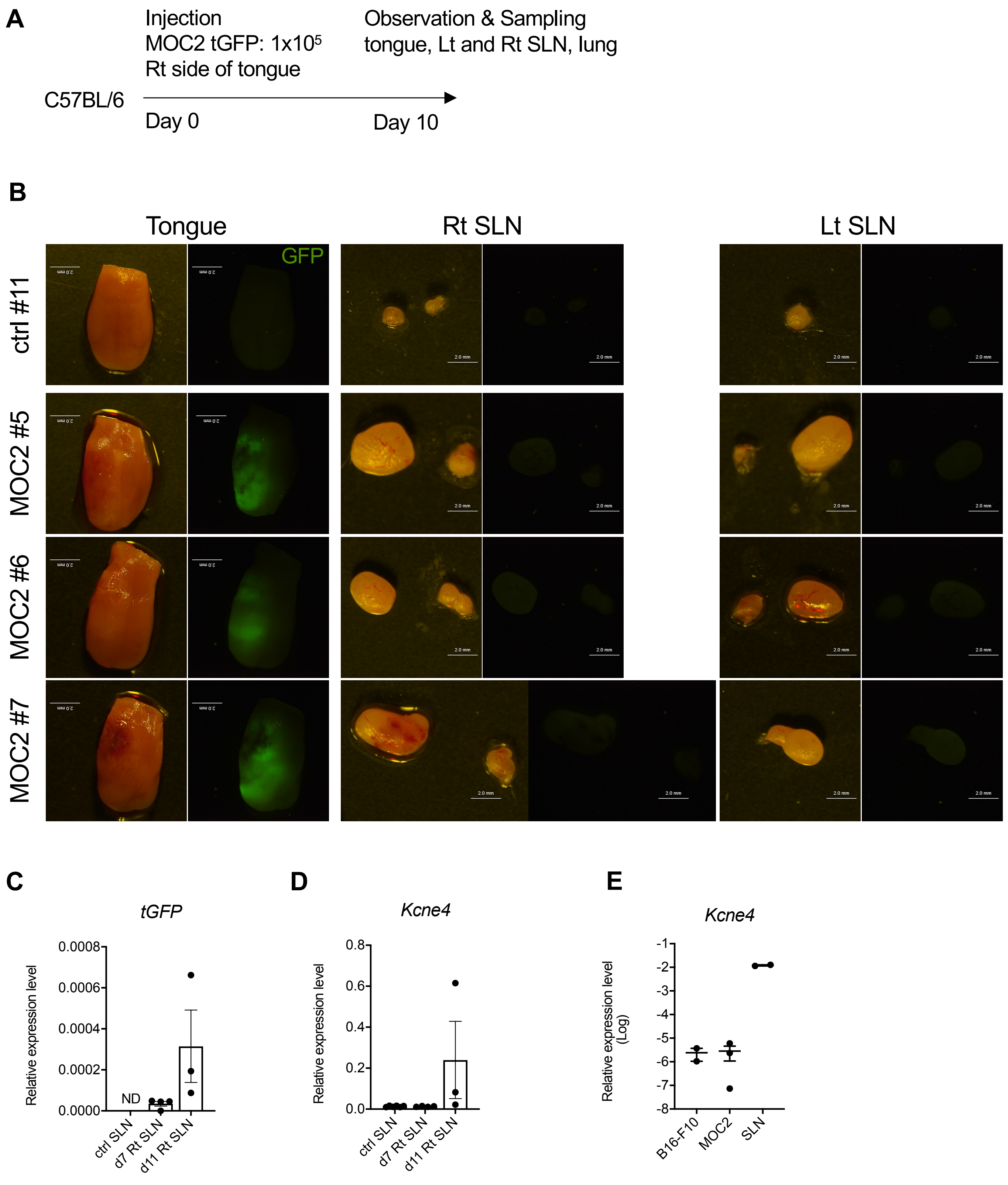

Supplementary Fig. S3. Induction of *Kcne4* in SLNs by orthotopic transplantation of oral squamous cell carcinoma. MOC2 is a metastatic oral SCC cell line, were modified to stably express turboGFP (tGFP) and were transplanted into tongue. (**A**) Experimental schedule of transplantation. (**B**) Macroscopic image of the tongue and SLN on day 7 after transplantation. Fluorescence of tGFP was detected in the tongue transplanted with MOC2-tGFP. (**C**) The expression level of *tGFP* mRNA was analyzed by qRT-PCR. *tGFP* mRNA was detected in SLNs 7 days after transplantation and increased on day 11. (**D**) *Kcne4* mRNA expression was increased in the right lymph node of mice 11 days after transplantation. Expression levels were normalized with *Actb*. (**E**) *Kcne4* expression levels in B16-F10 and MOC2.
